# Radiometric Measurement Comparison on the Integrating Sphere Source Used to Calibrate the Moderate Resolution Imaging Spectroradiometer (MODIS) and the Landsat 7 Enhanced Thematic Mapper Plus (ETM+)

**DOI:** 10.6028/jres.108.020

**Published:** 2003-06-01

**Authors:** James J. Butler, Steven W. Brown, Robert D. Saunders, B. Carol Johnson, Stuart F. Biggar, Edward F. Zalewski, Brian L. Markham, Paul N. Gracey, James B. Young, Robert A. Barnes

**Affiliations:** Laboratory for Terrestrial Physics, National Aeronautics and Space Administration, Goddard Space Flight Center, Greenbelt, MD 20771; National Institute of Standards and Technology, Gaithersburg, MD 20899-0001; Remote Sensing Group, Optical Sciences Center, University of Arizona, Tucson, AZ 85721; Laboratory for Terrestrial Physics, National Aeronautics and Space Administration, Goddard Space Flight Center, Greenbelt, MD 20771; Raytheon Santa Barbara Remote Sensing, Goleta, CA 93117; Science Applications International Corporation, Beltsville, MD 20705

**Keywords:** calibration, Earth Observing System (EOS), integrating sphere, Moderate Resolution Imaging Spectroradiometer (MODIS), Landsat 7 Enhanced Thematic Mapper Plus (ETM+), remote sensing, spectral radiometry, transfer radiometers

## Abstract

As part of a continuing effort to validate the radiometric scales assigned to integrating sphere sources used in the calibration of Earth Observing System (EOS) instruments, a radiometric measurement comparison was held in May 1998 at Raytheon/Santa Barbara Remote Sensing (SBRS). This comparison was conducted in support of the calibration of the Moderate Resolution Imaging Spectroradiometer (MODIS) and the Landsat 7 Enhanced Thematic Mapper Plus (ETM+) instruments. The radiometric scale assigned to the Spherical Integrating Source (SIS100) by SBRS was validated through a comparison with radiometric measurements made by a number of stable, well-characterized transfer radiometers from the National Institute of Standards and Technology (NIST), the National Aeronautics and Space Administration’s Goddard Space Flight Center (NASA’s GSFC), and the University of Arizona Optical Sciences Center (UA). The measured radiances from the radiometers differed by ±3 % in the visible to near infrared when compared to the SBRS calibration of the sphere, and the overall agreement was within the combined uncertainties of the individual measurements. In general, the transfer radiometers gave higher values than the SBRS calibration in the near infrared and lower values in the blue. The measurements of the radiometers differed by ±4 % from 800 nm to 1800 nm compared to the SBRS calibration of the sphere, and the overall agreement was within the combined uncertainties of the individual measurements for wavelengths less than 2200 nm. The results of the radiometric measurement comparison presented here supplement the results of previous measurement comparisons on the integrating sphere sources used to calibrate the Multi-angle Imaging SpectroRadiometer (MISR) at NASA’s Jet Propulsion Laboratory (JPL), Pasadena, CA and the Advanced Spaceborne Thermal Emission and Reflection Radiometer (ASTER) at NEC Corporation, Yokohama, Japan.

## 1. Introduction

The Earth Observing System (EOS) is an 18 year international multi-satellite, multi-instrument program in remote sensing of the Earth. The goal of the program is to advance the scientific understanding of the Earth system and its changes on a global scale. To achieve this goal, EOS instruments will make global, continuous, long time series radiance and reflectance measurements of the Earth. The first EOS satellite, Terra, launched in December 1999, is comprised of five instruments designed to monitor the Earth’s atmosphere, oceans, land, cryosphere, and their interaction with the incident solar radiation. The five EOS Terra instruments include the Moderate Resolution Imaging Spectroradiometer (MODIS) [1], the Advanced Spaceborne Thermal Emission and Reflection Radiometer (ASTER) [2], the Multi-angle Imaging SpectroRadiometer (MISR) [3], the Clouds and the Earth’s Radiant Energy System (CERES) instrument [4], and the Measurements of Pollution in the Troposphere (MOPITT) instrument [5]. On orbit, these instruments make geolocated radiance and reflectance measurements that will be combined to form the basis for a multidisciplinary study of the Earth system. The resulting radiance and reflectance images from these instruments will be formed into a number of geophysical products. The Landsat 7 satellite, also launched in December 1999, carries the Enhanced Thematic Mapper Plus (ETM+) instrument [6]. Measurements from ETM+ will be combined with those made from the EOS Terra instruments for regional process studies and models. The MODIS and CERES instruments are also on the EOS Aqua platform that was launched on May 4, 2002, effectively continuing the data set acquired by the EOS Terra instruments; the follow-on to the ETM+ instrument, the Landsat Data Continuity Mission (LDCM), is in preparation.

The capability for researchers to combine radiometric measurements and derived geophysical products from multiple EOS instruments on the same or different satellite platforms is essential. The accuracy of these analyses is critically linked to the pre-launch calibration of the instruments and the assessment of the post-launch calibration. Accurate radiometric calibration and characterization is by reference to a common set of recognized physical standards, good measurement practice, and realistic uncertainty budgets. With respect to the pre-launch calibration of the EOS Terra and Landsat 7 ETM+ instruments, a set of transfer radiometers operating in the visible through shortwave infrared wavelength region (from 400 nm to 2500 nm) has been independently developed and calibrated by several metrology laboratories and used to assess standard sources of spectral radiance. These laboratories include the National Institute of Standards and Technology (NIST), the National Aeronautics and Space Administration’s Goddard Space Flight Center (NASA’s GSFC), the University of Arizona Optical Sciences Center (UA), and the National Metrology Institute of Japan/National Institute of Advanced Industrial Science and Technology—NMIJ/AIST (formerly the National Research Laboratory of Metrology—NRLM) in Tsukuba, Japan. Under the direction of the EOS Project Science Office, these radiometers make simultaneous, comparative measurements of the integrating sphere sources used to calibrate EOS instruments [7–10] to assess the accuracy of the radiometric values assigned to the integrating sphere sources by the EOS instrument builders. The transfer radiometers are calibrated by their home institution using radiometric standards maintained by the appropriate national standards laboratories. As part of the study, the radiometric stability and repeatability of the spheres are also determined.

In May 1998, NIST, UA, NASA’s GSFC, and Raytheon Santa Barbara Remote Sensing (SBRS) participated in a radiometric measurement comparison using the SBRS Spherical Integrating Source (SIS100). The SIS100 is the large aperture, uniform radiant source used to calibrate the EOS Terra and Aqua MODIS instruments and the Landsat 7 ETM+ instrument. The 1998 measurement comparison addressed the validation of the SBRS-assigned radiometric values, sphere repeatability, and sphere stability. During the comparison, measurements were made by the participating radiometers on 37 sphere radiance levels. This paper presents and discusses the results from that radiometric measurement comparison.

The results are relevant to the calibration and characterization of current and future satellite and ground instruments. The radiance calibration approaches employed by SBRS on the SIS100 and described in this paper are used by a number of other institutions. The results presented here constitute the first, careful examination and validation of those approaches. The SIS100 will be used in the pre-launch radiance calibration of the Visible Infrared Imaging Radiometer Suite (VIIRS) scheduled to fly on both the National Polar-orbiting Environmental Satellite System (NPOESS) and the National Polar-orbiting Environmental Satellite System Preparatory (NPP) projects. The stability, repeatability, and radiance measurement methodologies of the SIS100 examined in this paper will provide important guidance to SBRS in their use of the SIS100 in the calibration of VIIRS.

## 2. Instruments

### 2.1 Raytheon SBRS SIS100

Table 1 gives the specifications for the SIS100. For the radiometric measurement comparison described in this paper, the sphere was located in the Optics Lab of the SBRS facility in Goleta, California. The SIS100, manufactured by Labsphere, Inc.,^1^ is a hollow spun aluminum shell, coated on its interior surface with Spectraflect™, a barium sulfate-based coating. The sphere contains 37 quartz halogen lamps comprised of eighteen 200 W lamps, nine 45 W lamps, and ten 8 W lamps. Four power supplies are used to operate the lamps, with two power supplies for the 200 W lamps and one power supply each for the 45 W and 8 W lamps. Lamps powered by each supply are wired in series and are current-regulated to 0.03 %. The current through each lamp type is monitored using precision shunts and a scanning multimeter. Lamps which are shut off are replaced in the circuit with load resistors. The total operating time for each lamp is monitored using individual elapsed time meters. There are no temperature monitors on the SIS100.

To meet the MODIS spectral radiance calibration uncertainty specification of ± 5 % (*k* = 1), SBRS calculated that the spectral radiance calibration uncertainty of the SIS100 must be less than ± 3 % (*k* = 1). Table 2 shows the SBRS calculated relative standard uncertainties for the SIS100. The spectral regions are separated into visible (VIS), near infrared (NIR), and shortwave infrared (SWIR). In the SWIR column, the uncertainties for the irradiance standard refer to the result at 1600 nm; this component is larger at longer wavelengths. In support of the radiometric comparisons presented in this paper, the SIS100 was calibrated by SBRS in April 1998. The SBRS calibration employed a quartz halogen standard irradiance lamp and a pressed halon diffuse reflecting plaque with a known 0°/45° bidirectional reflectance factor (BRF). This produces a source of known spectral radiance. The spectral irradiance values for the SBRS lamp and the BRF values for the SBRS plaque were traceable to values disseminated by NIST. Two SBRS standard irradiance lamps were used, both calibrated by a commercial standards laboratory (Optronic Laboratories, Incorporated). The BRF of the plaque was determined at SBRS by comparison to a Spectralon™ standard that had been calibrated for SBRS by NIST. The NIST BRF values assigned to the Spectralon standard were based on absolute bi-directional reflectance measurements for the visible and near infrared (to 1700 nm); beyond 1700 nm the BRF values were determined from measurements of the 6° directional-hemispherical reflectance, assuming the ratio of the bi-directional to the directional-hemispherical reflectance was constant [11].

A modified Cary-14 spectroradiometer configured for radiance measurements viewed the lamp/diffuser source and compared the measured detector output to that measured while viewing the SIS100. For measurements from 360 nm to 1000 nm, a photomultiplier tube (PMT) detector was used. For measurements from 700 nm to 2300 nm, an indium antimonide (InSb) detector was used. The SIS100 spectral radiance at wavelength *λ*, for *n* lamps illuminated, *L_λ_*_,SIS_ (*n*), was calculated using Eq. (1)

$$L_{\lambda ,\text{SIS}}(n)=f_{r}E_{\lambda }\frac{V_{\lambda ,\text{SIS}}(n)}{V_{\lambda ,\text{STD}}}.$$ (1)

In Eq. (1) *f*_r_ is the spectral 0°/45° bidirectional reflectance distribution function of the halon plaque at wavelength *λ*, *E_λ_* is the spectral irradiance of the standard lamp, *V_λ_*_,SIS_ (*n*) is the signal from the measurement of the SIS100 at wavelength *λ* and with *n* lamps illuminated, and *V_λ_*_,STD_ is the signal from the measurement of the illuminated plaque.

Measurements were made at the wavelengths in the standard lamp test report (at 20 wavelengths from 360 nm to 2300 nm), and the measurement results were calculated at 1 nm and 2 nm intervals using cubic-spline interpolation. The calibration of the SIS100 was performed three times in the visible/near infrared and twice in the shortwave infrared, and the results of the individual calibrations were compared for consistency and repeatability.

For the April 1998 SIS100 calibration, the sphere was operated by Raytheon SBRS in the following manner. SBRS initially measured the sphere radiance with all 200 W, 45 W, and 8 W lamps illuminated. Radiance measurements continued as individual 200 W lamps were successively extinguished, followed by individual 45 W lamps, followed by individual 8 W lamps. This procedure was repeated twice and the results were averaged. This multiple lamp wattage-type operation of the SIS100 was used in the calibration of the MODIS Aqua satellite instrument. A table with the SBRS calibration results was provided to the comparison participants before the start of the May comparison measurements.

The multiple lamp wattage-type operational approach differed from the approach used by SBRS prior to April 1998 in calibrating the SIS100 for the MODIS Terra and ETM+ satellite instruments. For those calibrations, measurements were performed with all lamps of one wattage type illuminated. Successive measurements were made after turning off individual lamps in that wattage type until only one lamp was illuminated. This process was repeated for all three lamp types, that is, with only lamps of the same type in operation at a particular time. Radiance values for the SIS100 lamp combinations used in the calibration of the MODIS Terra and ETM+ instruments were calculated by adding the results of the appropriate identical wattage measurements. SBRS and the comparison participants agreed that the multiple wattage-type operation should be adopted as the basis for the measurement comparison. However, it was also agreed that SIS100 radiances be measured for the identical wattage-type operation and a comparison of SIS100 radiances obtained through identical wattage-type and multiple wattage-type operational approaches should be performed.

**Fig. 1:**
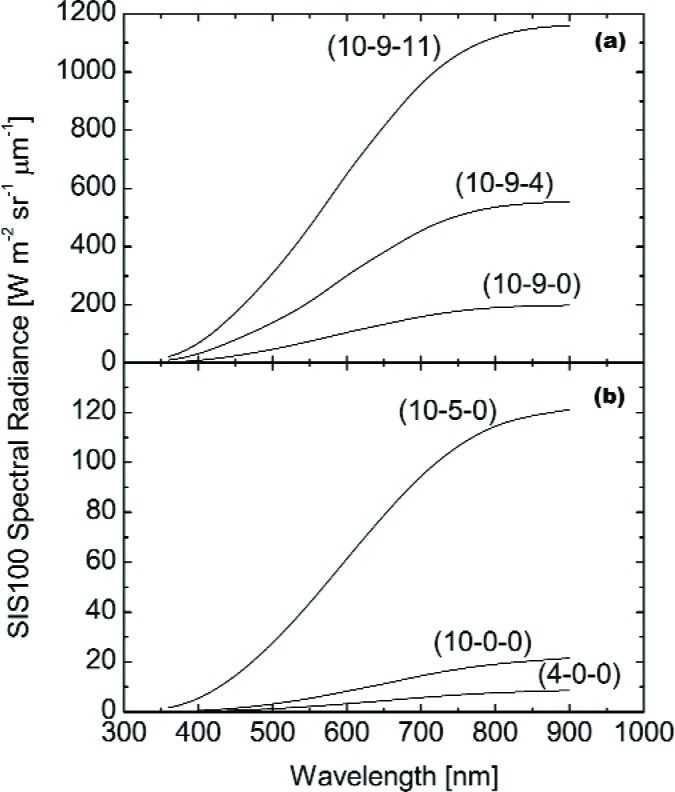
Spectral radiances for the SIS100 as determined by SBRS in the visible/near infrared for the principal radiance levels in the May 1998 comparison for (a) the three brightest levels and (b) the three dimmest levels.

**Fig. 2:**
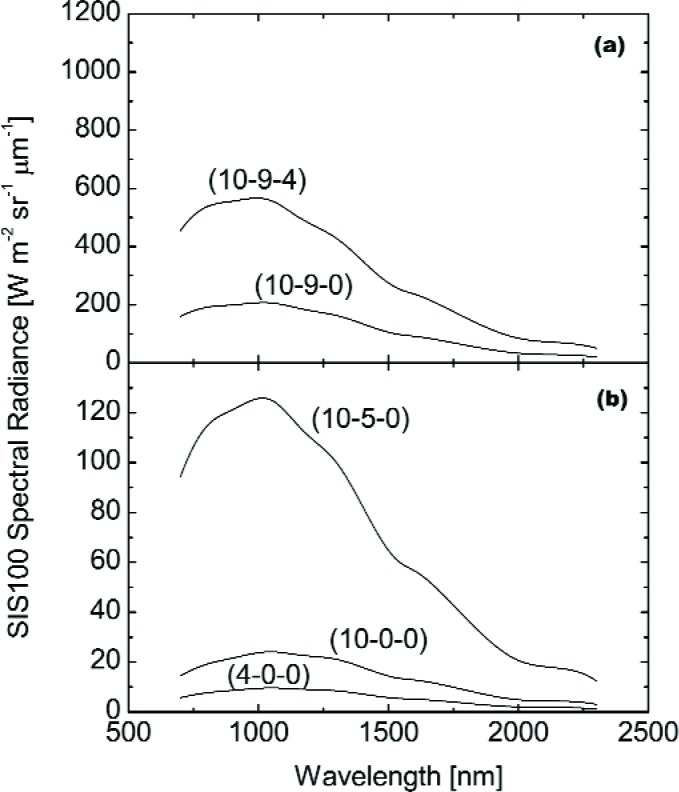
Spectral radiances for the SIS100 as determined by SBRS in the shortwave infrared for the principal radiance levels in the May 1998 comparison for (a) the two brightest levels (configuration (10-9-11) was not measured) and (b) the three dimmest levels.

The principal measurements in the May 1998 campaign were made at six SIS100 operating levels: (10-9-11), (10-9-4), (10-9-0), (10-5-0), (10-0-0), and (4-0-0). The values in parenthesis give the number and type of lamps illuminated in the sphere, with the lowest wattage listed first. For example, (10-9-11) refers to ten 8 W lamps illuminated, nine 45 W lamps illuminated, and eleven 200 W lamps illuminated. For the brightest level, (10-9-11), the SBRS values in the visible/near infrared extend from 360 nm to 900 nm in 1 nm intervals. For this level, there is no calibration in the shortwave infrared. For the other five levels, the SBRS calibration is from 360 nm to 1000 nm in 1 nm intervals and from 700 nm to 2300 nm in 2 nm intervals. The sphere spectral radiance curves from SBRS are shown in Figs. 1, and 2. Figure 1 gives the values from the visible/near infrared calibration. Figure 2 gives the values from the near and shortwave infrared calibration.

### 2.2 Transfer Radiometers

The five transfer radiometers in the May 1998 radiometric measurement comparisons are briefly described below.

#### 2.2.1 EOS/NIST Visible/Near Infrared Transfer Radiometer (VXR)

In the May 1998 radiometric comparisons, NIST participated with the EOS Visible/Near Infrared Transfer Radiometer (VXR) and the EOS Shortwave Infrared Transfer Radiometer (SWIXR). The VXR is a six channel filter radiometer of similar optical design to the SeaWiFS Transfer Radiometer (SXR) [12]. The VXR was built by NIST for the EOS Project Science Office, as was the SWIXR. In the May 1998 instrument comparison, both the VXR and SWIXR were calibrated and operated by NIST personnel. During the campaign, the VXR made radiometric measurements of the SIS100 and served as a sphere monitor (by off axis measurements) while other transfer radiometers were measuring the sphere output at normal incidence.

Table 3 gives the center wavelengths and bandwidths for the six VXR channels. The bandwidths Δ*λ* are calculated using Eq. (2),

$$\Delta \lambda =\int_{\lambda_{1}}^{\lambda_{2}}R_{\lambda }d\lambda$$ (2)

where *R_λ_* is the relative spectral response at wavelength *λ*, and the maximum value of *R_λ_* is normalized to unity. The relative spectral responsivities were determined using the NIST visible spectral comparator facility, which utilizes a lamp-illuminated prism-grating monochromator [13]. For all of the instruments in the May 1998 comparison, the bandwidths in Table 3 are roughly the same as the full width at half maximum (FWHM) values. The corresponding center wavelengths *λ*_C_ are calculated as weighted means, using Eq. (3),

$$\lambda_{C}=\frac{\int_{\lambda_{1}}^{\lambda_{2}}\lambda R_{\lambda }d\lambda }{\Delta \lambda }.$$ (3)

The VXR was calibrated in advance of the SBRS comparison using a NIST integrating sphere source, model number OL420, manufactured by Optronic Laboratories, Incorporated. At NIST’s request, a monitor photodiode was mounted in the sphere wall. In March 1998, the OL420 was calibrated for spectral radiance in NIST’s Facility for Automated Spectroradiometric Calibrations (FASCAL) [14]. In December 1999, revised calibration coefficients were applied to the VXR radiance measurements [15]. The relative standard uncertainties for the VXR in 1997 and 1998 are estimated to be 2 % for all but the 870 nm channel. For that channel, the uncertainty is estimated to be 3 % [16].

#### 2.2.2 Arizona Visible/Near Infrared Transfer Radiometer (UA VNIR)

The UA VNIR is a simple, single detector radiometer with a manual rotating filter wheel containing seven filters [17, 18]. Unlike the VXR, which has six complete optical and electronic channels and can make six measurements simultaneously, measurements with the UA VNIR are made sequentially by rotating the filter wheel. This manual operation is a trade off for the radiometer’s more simple optical and electronic design. The optical design is based on two precision apertures spaced at a known distance by invar rods. The filters are placed in front of the first aperture and a trap detector^2^ is placed immediately behind the second aperture [17]. With the removal of the front aperture, the transfer radiometer can measure irradiance calibration sources directly. With both apertures in place, the instrument functions as a standard radiometer. The seven UA VNIR channels correspond to several of the MODIS, ASTER, and MISR bands. Table 3 gives the center wavelengths and bandwidths for the channels in the UA VNIR [18]. The relative spectral responsivities were determined from measurements of the filter transmittances using a double monochromator.

The laboratory calibration of the UA VNIR also utilized lamp standards of spectral irradiance, in this case one lamp was calibrated by NIST (irradiance lamp number F330) and one was obtained from a commercial standards laboratory (irradiance lamp number F297 from Optronic Laboratories, Inc.). The principal (irradiance-mode) calibration of the instrument is made by viewing the irradiance standard with the front aperture removed [18]. A second calibration is performed by viewing a Spectralon™ plaque that is illuminated by the irradiance standard. The bidirectional reflectance distribution function (BRDF) of the plaque is measured in the calibration facility at the University of Arizona. The realization of reflectance at UA is based on a pressed polytetrafluoroethylene (PTFE) reflectance standard. This radiance-mode measurement is made with both apertures in place. The irradiance- and radiance-mode calibrations in the laboratory agree within 0.51 % (average of seven channels) with a standard deviation of 0.43 % and a worst-case difference of 1.4 % at 868 nm. The relative standard uncertainty estimate for the radiance-mode calibration is 2.1 % at 413 nm and 2.2 % at 667 nm [18].

#### 2.2.3 NASA/GSFC Landsat Transfer Radiometer (LXR)

The Landsat Transfer Radiometer (LXR) [19] is a six filter radiometer that shares the design of the SXR and VXR. The LXR was built by NIST for the Landsat Project Science Office. The wavelengths and bandwidths for the channels of the LXR are given in Table 3. The relative spectral responses for the first four LXR channels were configured to correspond to those for the Landsat instrument. Thus, there are four broad spectral response channels and two narrow ones, with the broad channels overlapping the narrow ones. The relative spectral responsivity of the six LXR channels was determined using a lamp-illuminated monochromator at GSFC [19].

The radiometric calibration of the LXR radiometer was performed by NIST using the OL420 source. As with the VXR, the source was calibrated using FASCAL. The LXR viewed the OL420 at a 1 m distance in April 1997 and January 1999; the OL420 was calibrated by FASCAL in October 1996, March 1998, November 1998 and May 1999. The two calibrations of the LXR agreed to within 1 %. The relative standard uncertainty in the transfer of the calibration from the FASCAL to the LXR was 0.5 % to 1 %, depending on the channel. The overall relative standard uncertainty of the LXR is 2 % to 3 %, also depending on the channel.

#### 2.2.4 Arizona Shortwave Infrared Transfer Radiometer (UA SWIR)

The UA SWIR [20] is a twelve channel filter radiometer employing a liquid nitrogen cooled InSb detector. As with the UA VNIR, the Arizona shortwave transfer radiometer uses two precision apertures to define the solid angle of its field-of-view. The UA SWIR channels are chosen to have center wavelengths corresponding with those in MODIS and ASTER. The UA SWIR channel at 1380 nm, which overlaps a water-vapor absorption feature, was designed for atmospheric studies, not transfer measurements of laboratory sources [21]. The wavelengths and bandwidths for the channels of the UA SWIR radiometer are given in Table 3. The radiometer also has two channels at wavelengths longer than 2300 nm that correspond with ASTER bands, channels that are not used in this comparison.

The UA SWIR is calibrated for spectral radiance responsivity in the laboratory using a standard irradiance lamp and a diffusely reflecting Spectralon™ plaque. The lamp and plaque are the same as those used in the calibration of the UA VNIR radiometer, so the assignment of the spectral irradiance and reflectance values, including their relationship to NIST standards, is as described in Sec. 2.2.2. For the UA SWIR radiometer, the plaque is placed 2.33 m from the standard lamp to provide calibration radiances for the radiometer that approximate the typical on-orbit radiances for the MODIS and ASTER instruments. The relative standard uncertainty for the UA SWIR is estimated to be between 3.3 % and 3.9 % [20].

#### 2.2.5 EOS/NIST Shortwave Infrared Transfer Radiometer (SWIXR)

For the May 1998 comparison, the SWIXR made measurements of the SIS100 at 29 wavelengths, in 50 nm wavelength intervals from 850 nm to 2250 nm. The SWIXR [22] is a scanning spectroradiometer that is built around a double monochromator. It uses all-reflective input optics and a liquid nitrogen cooled InSb detector. The operating wavelengths for the SWIXR range from 800 nm to 2500 nm, with a variable bandwidth that depends on the slit widths. For the May 1998 comparison, the slit widths of the SWIXR were kept constant and the bandwidth measurements ranged from 12.5 nm to 16.5 nm, depending on the wavelength (see Table 3).

The radiometric measurement comparison at SBRS in May 1998 was the first field deployment of the SWIXR. The SWIXR was calibrated at NIST immediately after the SBRS comparison in June 1998. This calibration was performed using an Optronic Laboratories, Incorporated OL450 integrating sphere source calibrated for spectral radiance using a variable temperature blackbody located in the NIST Radiance Temperature Laboratory [23]. For the SBRS deployment, the relative standard uncertainty of the SWIXR was 4.5 %. The SWIXR was also calibrated for wavelength before, during, and after the SBRS comparison using pen-ray emission lamps.

## 3. Band Averaged Spectral Radiances

Since the transfer radiometers have finite bandwidths, their measurement results are presented as band-averaged spectral radiances, *L*_B_, which are calculated as weighted averages of the spectral radiance at wavelength *λ*, *L*_λ_

$$L_{B}=\frac{\int_{\lambda_{1}}^{\lambda_{2}}L_{\lambda }R_{\lambda }d\lambda }{\int_{\lambda_{1}}^{\lambda_{2}}R_{\lambda }d\lambda }=\frac{\int_{\lambda_{1}}^{\lambda_{2}}L_{\lambda }R_{\lambda }d\lambda }{\Delta \lambda }.$$ (4)

In Eq. (4), the values *L_λ_* values are either from SBRS (see Figs. 1 and 2) or the spectral radiance standard used to calibrate the transfer radiometer. The *R_λ_* values are from the characterization of the transfer radiometers; they were provided by each participant. The actual calculation is performed as a summation. Therefore *L*_B_ corresponds to the expected band-averaged spectral radiances for each channel.

The wavelength spacings provided by SBRS in the spectral radiance curves do not match the wavelength spacings for the transfer radiometers. For the SBRS visible/near infrared spectral radiances (*L_λ_*’s), the results are given at every nanometer from 360 nm to the upper limit of the measurements, either 900 nm or 1000 nm (depending on lamp configuration in the SIS100). For the SBRS shortwave infrared spectral radiance curves, the spacing is every 2 nm from 700 nm to 2300 nm. For the channels of the two UA instruments and of the EOS SWIXR, the wavelength intervals for the spectral responses (*R_λ_*’s) are 0.1 nm. For the EOS VXR channels, the wavelength spacings are 0.5 nm.

To allow calculations of band-averaged spectral radiances using Eq. (4), the SBRS spectral radiances were matched to the wavelength intervals of the transfer radiometers using cubic spline interpolation. For the SBRS visible/near infrared spectral radiances, the interpolation is done in two parts—from 360 nm to 600 nm and from 600 nm to the upper limit for the measurements. Each interpolation covers a wavelength range of 400 nm or less. For the shortwave infrared, the interpolation contains four parts, each covering 400 nm. The interpolation routine for these calculations comes from the commercially-available Kaleidagraph data analysis and graphing program. For large data sets, the spline interpolation in this analysis package does not match the values at all of the input data points. By limiting the number of data points in the interpolation, the differences of the calculated values and input values (at the input value wavelengths) are less than 0.1 % at all wavelengths. In the vast majority of the cases, the differences are 0.01 % or less. Thus, the interpolation adds negligibly to the uncertainties in this comparison.

## 4. The May 1998 Radiometric Measurement Comparison

The radiometric measurement comparison was held from May 12 through 15, 1998 and was designed to address the issues of validation of SBRS-assigned sphere radiances, sphere stability and repeatability, and linearity of sphere output. Participants began arriving at Raytheon SBRS and setting up equipment during working hours on May 11. The equipment was allowed to warm-up overnight. SBRS provided their radiance data for the SIS100, which were measured in April 1998 in advance of the radiometric measurement comparison.

In addition to the these goals, as explained in Sec. 2.1 we wanted to examine the validity of the SBRS identical-wattage method of assigning spectral radiance values, which assumes separate measurements for configurations utilizing lamps of the same wattage (either 200 W, 45 W, or 8 W), when added are the same as the spectral radiances when all of the corresponding lamps are illuminated. Stated symbolically as an example for the 45 W and 8 W lamps, does *L_λ_*, _SIS_(*n*-0-0) + *L_λ_*, _SIS_(0-*m*-0) = *L_λ_*, _SIS_(*n-m*-0)? In all cases, the same individual lamps were utilized.

On May 12 and 13, the SBRS assigned sphere radiances were validated for the six principal lamp configurations; these involved lamps of different wattages (see Figs. 1 and 2). SIS100 operating levels (10-9-11), (10-9-4), (10-9-0), (10-5-0), (10-0-0), and (4-0-0) were measured by the transfer radiometers, and some levels were measured twice. When not directly measuring the SIS100, the EOS VXR (all six channels) and the UA SWIR (at 1.6 µm) monitored the stability of the sphere off-axis. Preliminary results from the radiometers were presented at the end of each day. The measurement schedule for the six principal levels is shown in Table 4.

**Fig. 3:**
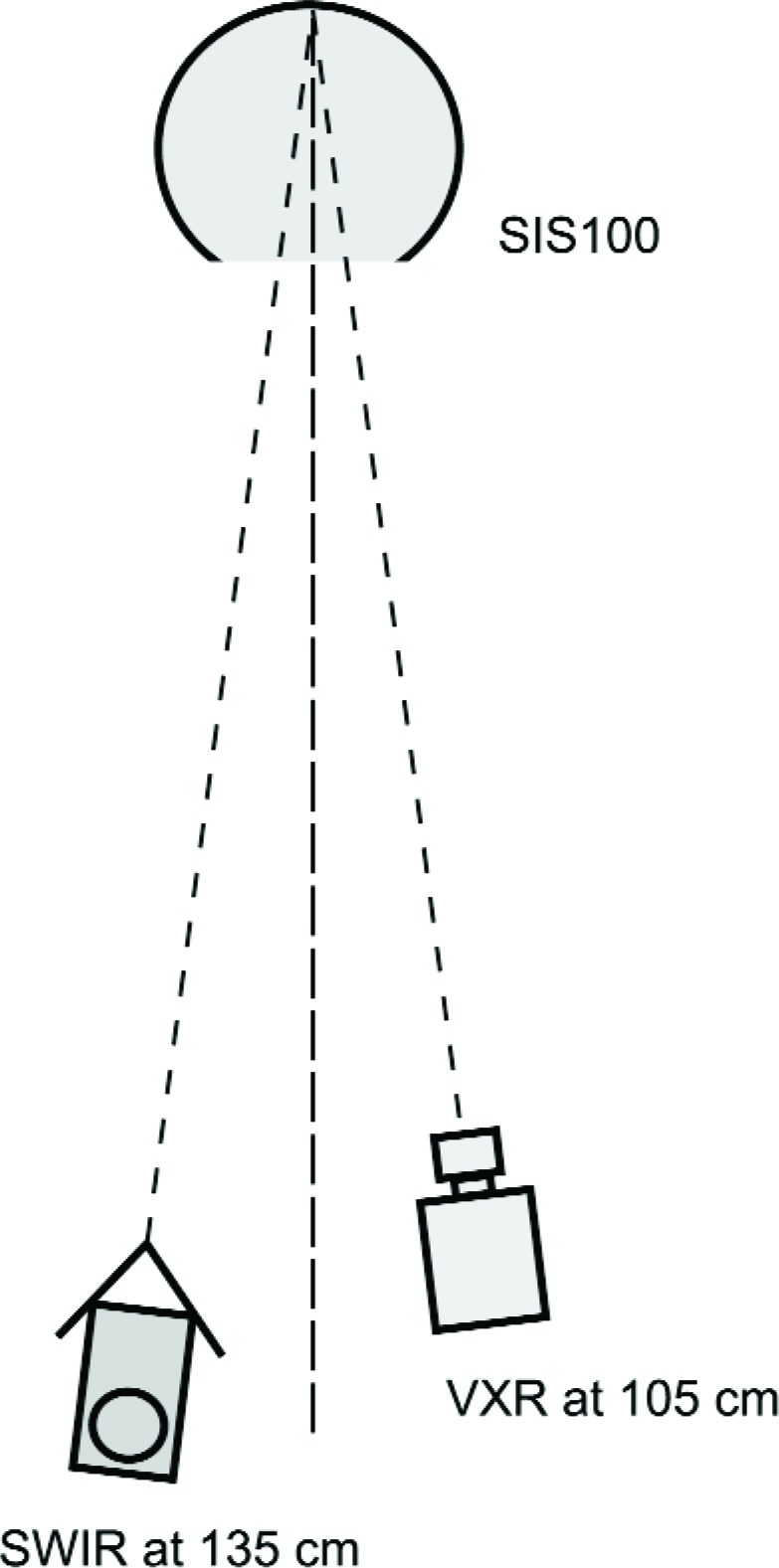
Schematic showing the positioning of the EOS VXR and the UA VNIR on May 14 and 15 for the off-axis monitoring of the SIS100.

On May 14, the EOS VXR and the UA SWIR were positioned slightly off-axis in front of the SIS100 and viewed an area in the back of the sphere corresponding to the area viewed by MODIS during its calibration. The positioning of the VXR and the UA SWIR for these measurements is shown in Fig. 3. In this configuration, the VXR and the UA SWIR radiometers repeated the complete April 1998 SBRS calibration of the sphere by measuring 33 of the 37 possible lamp configurations, starting with (10-9-14) as the brightest level. Upon completion of this study, seven different 8 W lamps were operated individually to examine their repeatability, since these levels were included in the set of 33. On May 15, the remaining four of the 37 possible levels were measured, along with nine selected 200 W lamp levels and five selected 45 W lamp levels, using the VXR and the UA SWIR radiometers.

## 5. Results

The comparison results are presented in terms of the difference, in percent, of the measurement by the transfer radiometer from a calculated value. The calculated values are obtained using Eq. (4), the spectral radiance curves provided by SBRS, and the relative spectral responses for the transfer radiometers. The measurements made by the transfer radiometers, which result in a net signal, are related to band-averaged spectral radiances through the measurement equation. Before the SBRS measurements, each transfer radiometer was calibrated in its laboratory using its own calibrated radiance source and the radiometric calibration coefficient for each radiometer channel was derived using Eq. (4), the laboratory spectral radiance curve, and the relative spectral response for that channel. Specifically, the measured band-averaged spectral radiances are equal to $\frac{S_{\text{SIS}}}{S_{\text{CAL}}}L_{B,\text{CAL}}$, where *S* is the net signal for a channel of a transfer radiometer and the subscript “cal” refers to calibration of the transfer radiometer. Therefore, the measurements at SBRS are a comparison of the spectral radiance values of the calibration sources with the SIS100 values, assuming that the radiometric calibration coefficients and spectral responsivities of the transfer radiometers are constant.

### 5.1 Comparisons at the Six Lamp Levels

The primary purpose of the comparison measurements is the validation of the SBRS-assigned sphere radiances. This is accomplished through comparisons at six lamp levels that span the sphere levels from bright, (level 10-9-11) to dim, (level 4-0-0).

#### 5.1.1 Comparisons in the Visible/Near Infrared

**Fig. 4:**
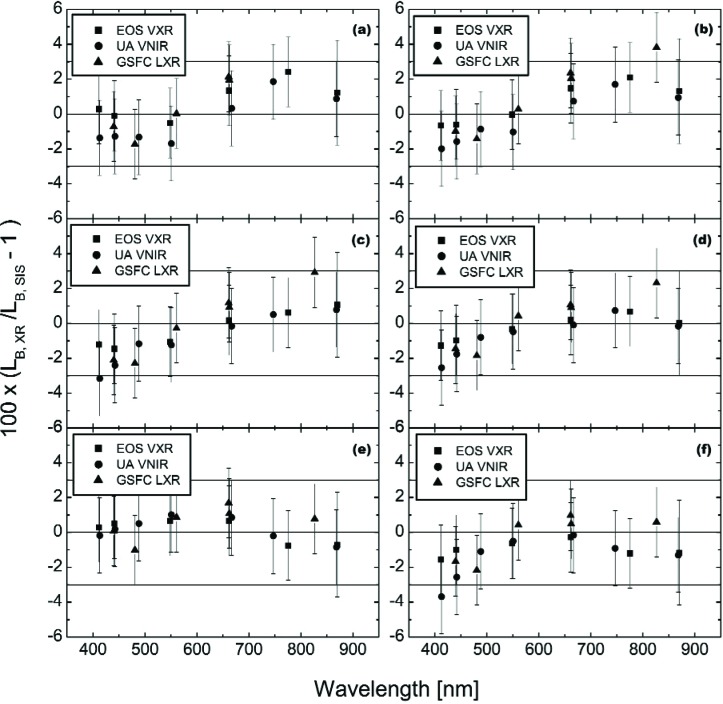
Results of the visible and near infrared radiometer measurements of the SIS100, shown as the percent difference of the band-average radiances measured by the transfer radiometers *L*B, XR with the band-averaged SIS100 radiances *L*B, SIS as the reference, for the six principal SIS100 levels: (a) (10-9-11); (b) (10-9-4); (c) (10-9-0); (d) (10-5-0); (e) (10-0-0); and (f) (4-0-0).

Figure 4 shows the comparison results for the three visible/near infrared radiometers for the on axis measurements of lamp configurations (10-9-4), (10-9-0), (10-5-0), acquired on May 12, and (10-9-11), (10-0-0), (4-0-0), acquired on May 13. The two VXR data sets (see Table 4), taken at the beginning and end of the measurement sequence, were averaged. The results are presented as the percent difference of the measurements by the radiometers from the expected spectral radiances calculated using the SBRS spectral radiance curves and the relative spectral responses of the radiometer channels. The figure includes the uncertainties associated with the calibration of the radiometer. It also includes three horizontal reference lines, corresponding to differences from SBRS of 3 %, 0 %, and −3 %. Recall that the requirement for the MODIS instrument is a relative standard uncertainty of 5 %.

As described above, the SBRS calibration of the (10-9-11) lamp level did not extend beyond 900 nm. This level and the others between (10-9-18) to (10-9-5) provide sufficient flux to calibrate satellite instruments in the blue and green portions of the spectrum, from about 400 nm to 550 nm. For wavelengths beyond 900 nm, these levels produce more than the maximum spectral radiances to ever be observed by satellite instruments on orbit. This eliminates the need to calibrate the sphere into the shortwave infrared for these levels. The brightest lamp level with a SBRS calibration past 900 nm is (10-9-4), and it was included for study in this comparison.

With its relatively broad bandwidth of 109.3 nm, the 827.0 nm channel of the GSFC LXR has significant response for wavelengths from 900 nm to 950 nm. Since SBRS provided no spectral radiance values for the SIS100 in this spectral range for the (10-9-11) level, the band-averaged spectral radiance cannot be calculated for this LXR channel, and consequently this data point is missing from Fig. 4a.

For 110 of the 113 measurement results in Fig. 4, the results are within 3 % of the SBRS-based values. For most of the measurements in Fig. 4, the *k* = 1 uncertainties, illustrated using the vertical lines, intersect the line at zero difference. For the complete ensemble of comparison points in Fig. 4, the mean difference of the measured values from the SBRS calibration curve is −0.1 % with a standard deviation of 1.3 %, illustrating good agreement.

**Fig. 5:**
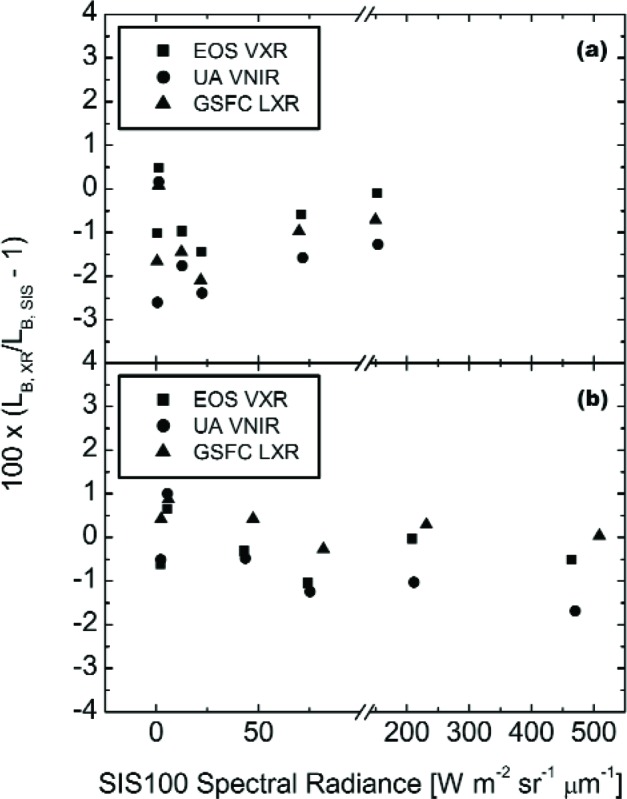
Results from Fig. 4 plotted versus sphere radiance for two spectral regions: (a) near 440 nm, and (b) near 550 nm. For each transfer radiometer, the abscissa gives the calculated radiance, based on the SBRS values (note the scale break). These radiances correspond to the six principal SIS100 lamp configurations, from (10-9-11), the brightest, to (4-0-0), the dimmest.

**Fig. 6:**
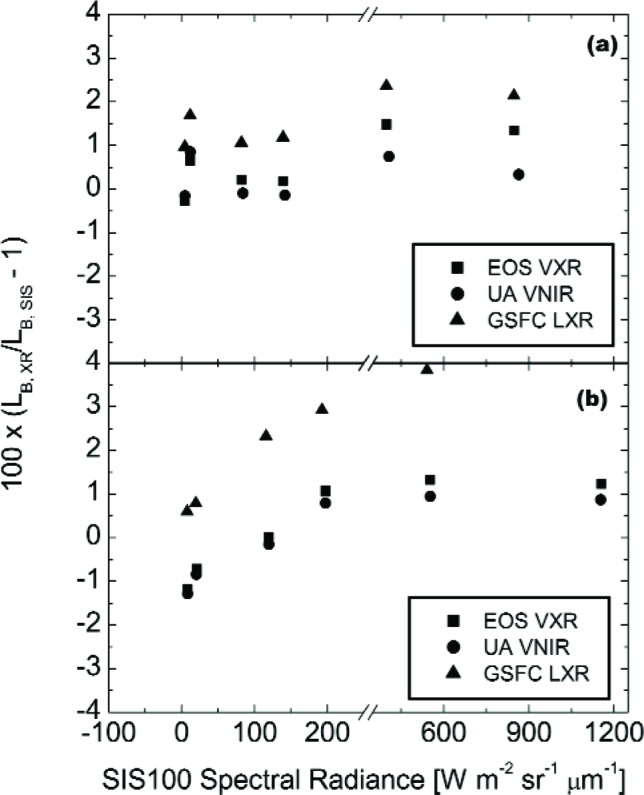
Results from Fig. 4 plotted versus sphere radiance for two spectral regions: (a) near 660 nm, and (b) near 870 nm. For each transfer radiometer, the abscissa gives the calculated radiance, based on the SBRS values (note the scale break). These radiances correspond to the six principal SIS100 lamp configurations, from (10-9-11), the brightest, to (4-0-0), the dimmest. For the GSFC LXR, there is no comparison for the 827 nm channel (see text for details).

Figure 5 shows the results of Fig. 4 plotted versus sphere spectral radiance corresponding to two spectral regions: (a) near 440 nm, and (b) near 550 nm. Figure 6 is similar except the results at two other spectral regions are shown: (a) near 660 nm, and (b) near 870 nm. As with Fig. 4, we show the relative difference of the measured values from the expected ones. The six sphere radiance levels correspond to lamp configuration (10-9-11) to (4-0-0). As explained above, the LXR results at 870 nm for level (10-9-11) are not reported (Fig. 6b).

At least a portion of the relative differences among the three radiometers at each SIS100 radiance level are due, in all likelihood, to differences in the calibration of each radiometer. For each spectral grouping in Figs. 5 and 6, such differences arising from transfer radiometer calibration bias would be the same for each SIS100 level if the radiometers are linear,^3^ and there is no bias in the spectral radiance values for the different levels as provided by SBRS, either as a function of SIS100 level or versus wavelength for a particular level. The latter possibility exists because the radiometer channels compared are not spectrally identical, with different center wavelengths and bandwidths, see Table 3.

The results in Figs. 5 and 6 confirm that there are relative offsets among the radiometers that are relatively independent of SIS100 radiance level. For example, the GSFC LXR is high compared to the other two radiometers for three of the four spectral regions, with the most serious discrepancy at its 827 nm channel. However, the results also demonstrate a tendency for the three radiometers to shift as a group as a function of the SIS100 configuration measured, by up to 1 % or 2 %. For the 440 nm band, this is evident at the dimmer SIS100 radiance levels, see Fig 5a. This result imposes a limitation on using the SIS100 for linearity characterization, as recognized previously by SBRS.

#### 5.1.2 Comparisons in the Shortwave Infrared

**Fig. 7:**
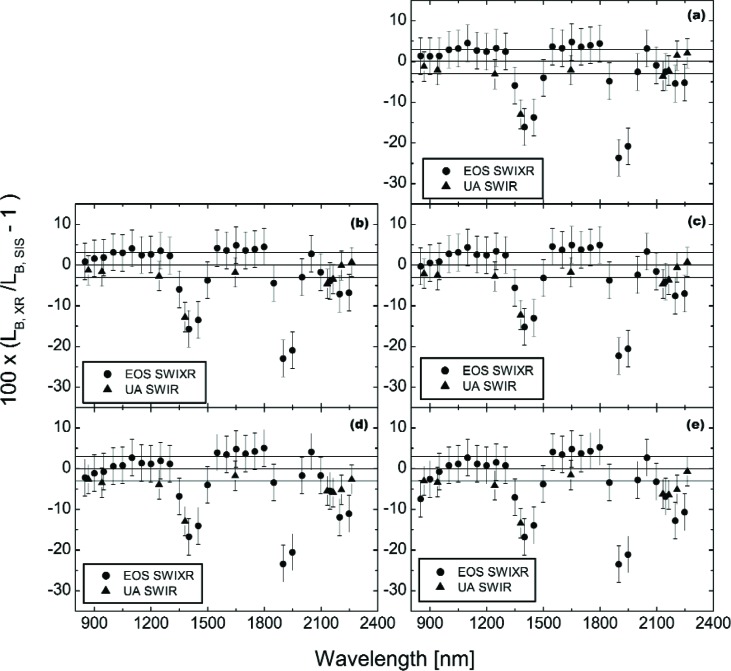
Results of the shortwave infrared radiometer measurements of the SIS100, shown as the percent difference of the band-average radiances measured by the transfer radiometers *L*B, XR with the band-averaged SIS100 radiances *L*B, SIS as the reference, for the six principal SIS100 levels: (a) (10-9-4); (b) (10-9-0); (c) (10-5-0); (d) (10-000); and (e) (4-0-0).

Figure 7 shows the comparison results for the two shortwave infrared radiometers in a format similar to the comparisons in Fig. 4. The figure includes the relative standard uncertainties associated with the calibration of the radiometer as vertical lines. It also includes three horizontal reference lines, one at 3 % difference from SBRS, one at zero difference, and one at −3 % difference; again these limits were selected by SBRS in order to meet the MODIS requirement of 5 %. In Fig. 7, the comparisons extend from 850.2 nm, the shortest wavelength of the EOS SWIXR, to 2263.0 nm, the longest wavelength of the UA SWIR.

**Fig. 8:**
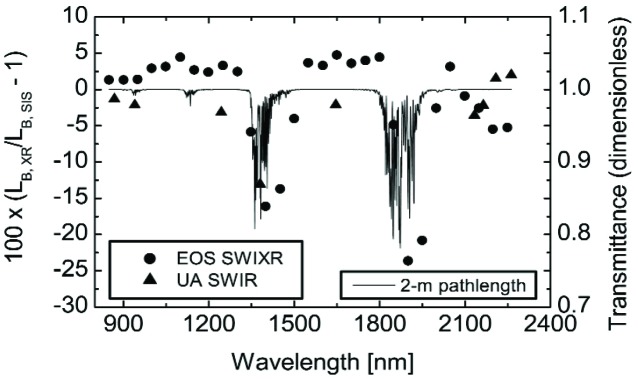
The results of Fig. 7a overlaid with the atmospheric transmittance for a 2 m path through the atmosphere, with conditions of 1 × 10^5^ Pa, 25 °C, and 30 % relative humidity. In the model, the absorption is due to water and carbon dioxide.

Figure 8 shows the comparison results from Fig. 7a, the (10-9-4) level, overlaid with the calculated atmospheric transmittance for a 2 m pathlength and conditions approximating those in the laboratory. The transmittances are calculated at 1 nm intervals using the MODTRAN [24] model, a pressure of 1 × 10^5^ Pa, a temperature of 25 °C, and a relative humidity of 30 %. No attempt is made to apply the atmospheric transmittances as corrections to the comparison results; however, there is strong evidence to link the atmospheric effects to the large comparison differences near 1350 nm and 1850 nm. There are two issues to consider related to atmospheric absorption. First, the NIST-assigned values of spectral irradiance are selected to avoid these regions, which results in calibration values at 800 nm, 900 nm, 1050 nm, 1150 nm, 1200 nm, 1300 nm, 1540 nm, 1600 nm, 1700 nm, 2000 nm, 2100 nm, 2300 nm, and 2400 nm. Interpolation by SBRS to values within regions of atmospheric absorption results in an overestimate of the radiance of the SIS100. Second, the atmospheric conditions of the relevant optical paths during each step in the measurement chain (calibration of the SIS100, calibration of the radiometers, and measurements during this study) most likely differ, which results in variability. For the SIS100, the optical pathlength inside the sphere depends on the wall reflectance and conditions inside the sphere, which may depend on thermal history.

A cursory examination of Fig. 8 shows the differences with the EOS SWIXR and the UA SWIR radiometers have the expected behavior within regions of atmospheric absorption, except they appear to be offset spectrally by about 50 nm. This discrepancy may be related to the abovementioned variability or the use of smooth interpolation through these critical regions (1300 nm to 1540 nm and 1700 nm to 2000 nm), which might result in spectrally dependent biases.

**Fig. 9:**
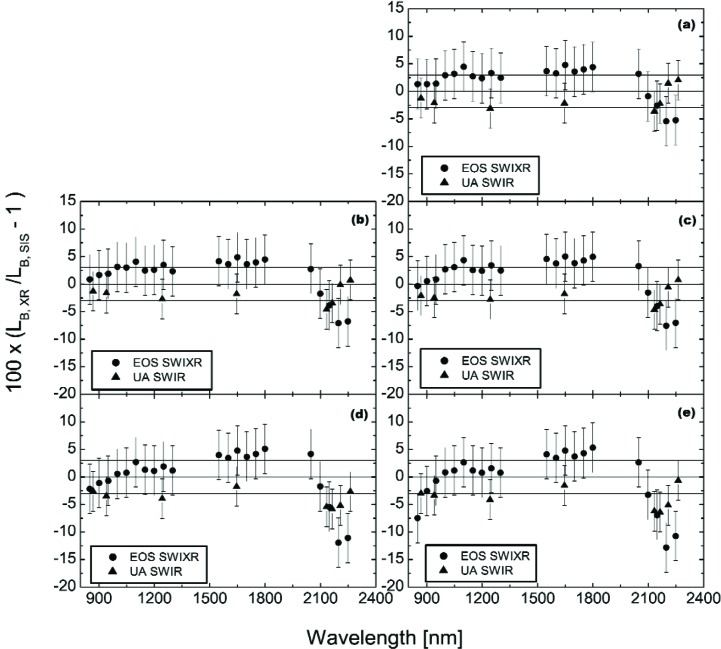
Results of the radiometer measurements of the SIS100 for the six principal levels: (a) (10-9-4); (b) (10-9-0); (c) (10-5-0); (d) (10-0-0); and (e) (4-0-0). The results are as in Fig. 7, except that measurements in the atmospheric absorption intervals—1350 nm to 1500 nm and 1850 nm to 2000 nm—have been removed.

As a result of these issues, we have removed the EOS SWIXR measurements from 1350 nm to 1500 nm and from 1850 nm to 2000 nm from the comparison results. In addition, we have removed the UA SWIR measurement at 1380 nm from the comparison results. The revised comparison data set is shown in Fig. 9.

The level of agreement can be described by noting that for the data in Fig. 9, the majority (131 of 150) agree with SBRS for *k* = 1 (uncertainties illustrated using the vertical lines in Fig. 9). For wavelengths from 800 nm to 2100 nm, the spectral radiance values provided by SBRS lie—for the most part—between the measured values from the EOS SWIXR and the UA SWIR, with the EOS SWIXR results consistently higher than the UA SWIR. At 2200 nm and 2250 nm, the EOS SWIXR measurements range from 5 % to 12 % lower than the calculated values. In this wavelength region, the UA SWIR measurements are also consistently lower than the SBRS-derived radiances, but they are consistently higher than those from the EOS SWIXR. The increase of the magnitude of the overall discrepancy between the transfer radiometers and SBRS for wavelengths beyond 2100 nm is interesting. However, only a few these results fall outside the combined expanded uncertainty (*k* = 2).

**Fig. 10:**
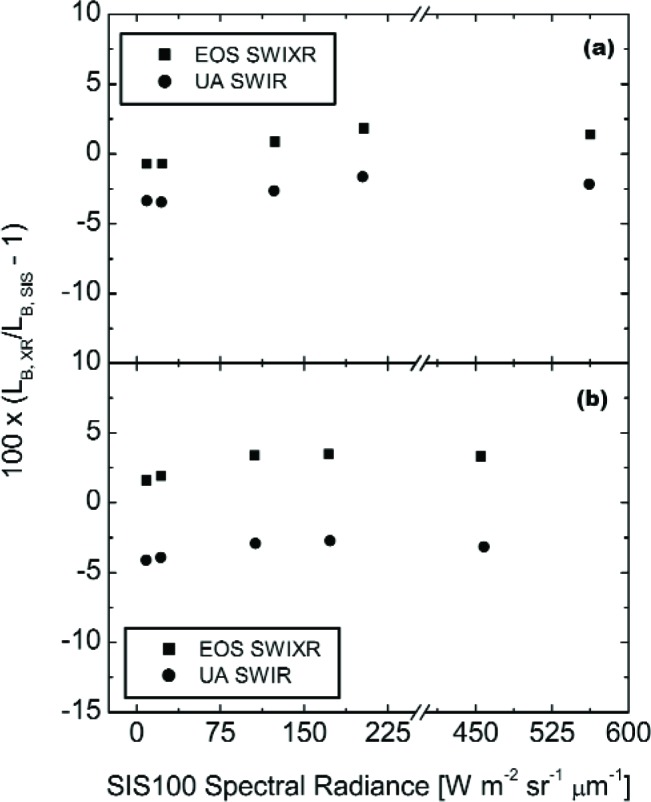
Results from Fig. 9 plotted versus sphere radiance for two spectral regions: (a) near 950 nm, and (b) near 1250 nm. For each transfer radiometer, the abscissa gives the calculated radiance, based on the SBRS values (note the scale break). These radiances correspond to the five principal SIS100 lamp configurations, from (10-9-4), the brightest, to (4-0-0), the dimmest.

**Fig. 11:**
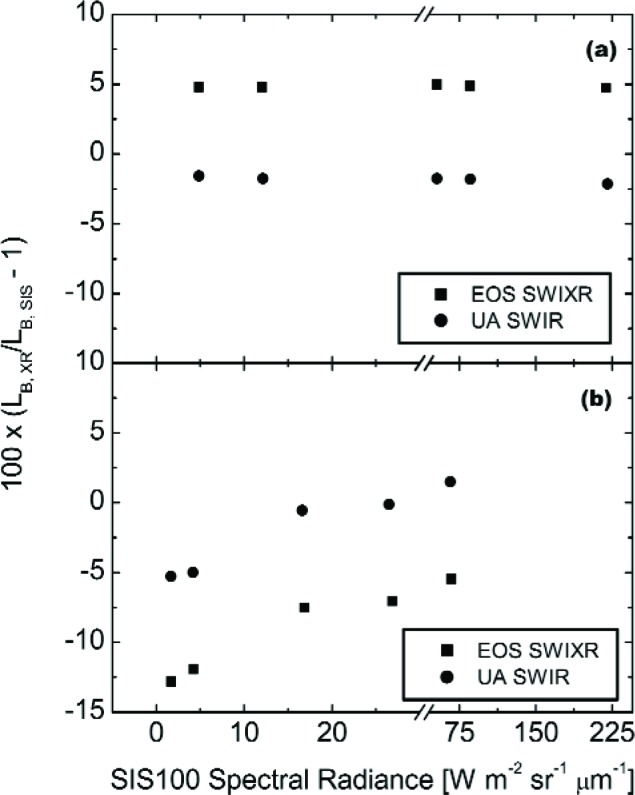
Results from Fig. 9 plotted versus sphere radiance for two spectral regions: (a) near 1650 nm, and (b) near 2200 nm. For each transfer radiometer, the abscissa gives the calculated radiance, based on the SBRS values (note the scale break). These radiances correspond to the five principal SIS100 lamp configurations, from (10-9-4), the brightest, to (4-0-0), the dimmest.

Figure 10 shows the results of Fig. 9 plotted versus sphere spectral radiance corresponding to two spectral regions: (a) near 950 nm, and (b) near 1250 nm. Figure 11 is similar except the results at two other spectral regions are shown: (a) near 1650 nm, and (b) near 2200 nm. As with Fig. 9, we show the relative difference of the measured values from the expected ones. The five sphere radiance levels correspond to configuration (10-9-4) to configuration (4-0-0), from bright to dim, respectively.

For the first three spectral regions (950 nm, 1250 nm, and 1650 nm), the differences of the two radiometers from the SBRS-derived values are nearly independent of the SIS100 radiance. At these wavelengths, the EOS SWIXR radiances are consistently higher than those from the UA SWIR, with differences ranging from 3 % to 7 %. If the transfer radiometers are linear,^4^ the results indicate the spectral radiances assigned by SBRS do not exhibit a bias that depends on brightness level. This statement does not apply to the SBRS-measured radiances at 2200 nm, where the relative differences between the two radiometers track well, but compared to SBRS the differences depend on the radiance level of the SIS100. The change is about 7 % over this range, with the SBRS measurements showing increasing radiances, relative to the transfer radiometers, at the dimmer sphere levels.

#### 5.1.3 Comparisons in the Overlap Region for the Visible/Near Infrared and Shortwave Infrared Calibrations of the Sphere

As shown in Table 3, there are nearly similar wavelengths for the two UA radiometers and the two EOS instruments. Each of the Arizona radiometers has measurement wavelengths near 747 nm and 868 nm. For the EOS transfer radiometers, the SWIXR’s measurements at 850.2 nm and 900.4 nm bracket that from the VXR at 869.9 nm. This allows for a closer look at the results with radiometers from the same institution.

For the UA transfer radiometers, the differences from the SBRS-based radiances are shown in Table 5. Using the SBRS-based values as common references, the measurements from the UA SWIR are consistently lower than those from the UA VNIR both at 747 nm and 868 nm. At 747 nm, the UA SWIR results range from 0.55 % to 0.94 % lower than those from the UA VNIR, and at 868 nm the results range from 1.06 % to 2.27 % lower. These differences, while they may represent an unidentified source of bias, are well within the combined standard uncertainties for the two instruments.

For the EOS VXR and the EOS SWIXR, the differences from the SBRS-based radiances are shown in Table 6. For these instruments, the wavelength agreement is not as close as for the Arizona radiometers, with the VXR wavelength located approximately in the middle of the 50 nm spacing between the two SWIXR wavelengths. This limits the applicability of the SBRS-based values as common references. However, at 850 nm, the SWIXR values range from 0.19 % higher to 6.28 % lower than the corresponding VXR measurements at 870 nm. The greatest difference occurs at the lowest light level in the measurement set (4-0-0). This wavelength and this radiance level mark the limit for the SWIXR measurements at SBRS in May 1998, since the SWIXR was designed for measurements at 900 nm and longer. For the next highest radiance level in the comparison (10-0-0), the SWIXR measurements are 1.45 % lower than those for the VXR at 870 nm, well within the combined standard uncertainties for the two instruments. At 900 nm, the SWIXR values range from 0.58 % higher than those from the VXR at 870 nm to 1.37 % lower than those from the VXR—again, well within the combined standard uncertainties for the two instruments.

### 5.2 Measurements of the 37 Multiple Lamp Levels

On May 14, the EOS VXR and the UA SWIR instruments were placed in front of the SBRS integrating sphere (see Fig. 3), and the SIS100 was configured with fourteen 200 W, nine 45 W, and ten 8 W lamps illuminated. Measurements were made of the radiance in the exit aperture as lamps were extinguished one at a time. The 200 W lamps were extinguished in sequence, followed by the 45 W lamps and the 8 W lamps. On May 15, the sphere was configured with eighteen 200 W, nine 45 W, and ten 8 W lamps illuminated. For these measurements, the 200 W lamps were extinguished in sequence until fourteen 200 W lamps remained illuminated. The May 15 measurements completed the set of multiple lamp levels and repeated the measurement for the initial lamp configuration (10-9-14) that was done on May 14.

**Fig. 12:**
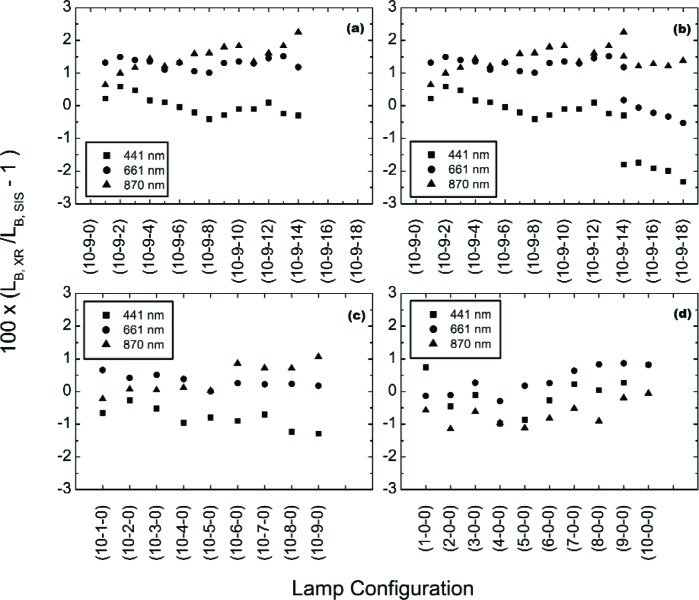
EOS VXR results of the off-axis measurements of the SIS100 for configurations where lamps of the same wattage were turned off sequentially. The measurements began on May 14 with 14 of the 200 W lamps, all of the 8 W lamps, and all of the 45 W illuminated, see (a); then individual 45 W lamps were extinguished, see (c); and then individual 8 W lamps were extinguished, see (d). In (b), we show the results for the four bright levels, (10-9-18) to (10-9-14), that were acquired on May 15 along with the May 14 results of the 200 W configurations.

The results of the May 14 and 15 off-axis measurements of the SIS100 using the EOS VXR are shown in Fig. 12. Figures 12a, 12c, and 12d show the results for the May 14 measurements, grouped by lamp wattage—the abscissa, given in terms of lamp configuration, should be nearly linear with radiance since the lamps are the same type. Figure 12b shows the 200 W measurements from both days. Results are shown for three of the EOS VXR channels, one in the blue, one in the red, and one in the near infrared. Comparison of these off-axis results to the on axis VXR results for the six principal levels shown in Figs. 5 and 6 gives good agreement, typically 0.5 %. We conclude that the SIS100 is an adequate Lambertian source for direct comparison of the off-axis EOS VXR and UA SWIR measurements to the SBRS-determined radiances. This was also observed previously, using the GSFC LXR [19].

For most of the measurements on May 14, the differences between the VXR and SBRS are reasonably constant, within ±0.5 %, with SIS100 lamp configuration for each measurement wavelength. However, when the (10-9-14) lamp configuration was repeated on the May 15, the results disagree by more than 0.5 %, see Fig. 12b. For this lamp configuration and particular measurement, the SIS100 was brighter (by 0.7 % at 870 nm, 1.0 % at 661 nm, and 1.5 % at 441 nm) on the second day.

**Fig. 13:**
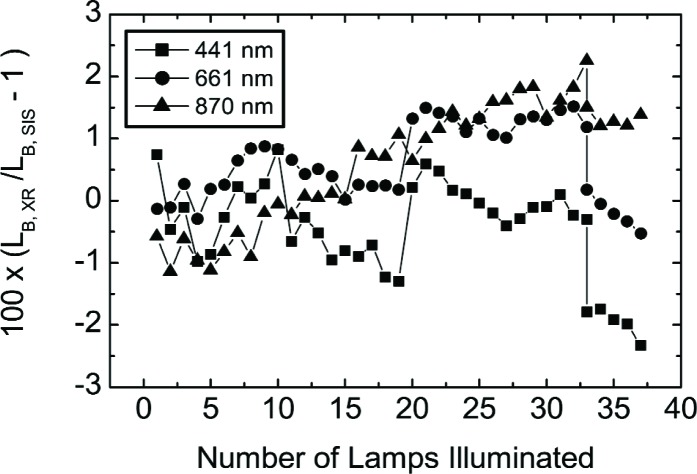
The results of Fig. 12 plotted as a function of number of lamps in the SIS100 that were illuminated for the measurements on May 14 and 15. The lines are to guide the eye. Configurations with one to nine 8 W lamps illuminated have abscissa values from 1 to 9; configurations with all 8 W and one to ten 45 W lamps illuminated have abscissa values from 10 to 19; and configurations with all 8 W, all 45 W and one to eighteen 200 W lamps illuminated have abscissa values from 20 to 37.

Other features exist, and they are most evident by plotting the results in Fig. 12 as a function of the number of lamps illuminated, see Fig. 13. The general trend at 870 nm is the difference between the VXR and the SBRS values depends on SIS100 radiance—the radiance is overestimated for the dim levels and underestimated for the bright levels—the total change is about 2 %. For the May 14 data at 441 nm and 661 nm, results for configurations with lamps of the same wattage stand out—a 1.5 % to 2 % effect at 441 nm when the last 200 W or the last 45 W lamps was extinguished. A likely explanation is thermal effects, since the operation intervals during the calibration of the SIS100 most likely differ from those on May 14 and 15.

**Fig. 14:**
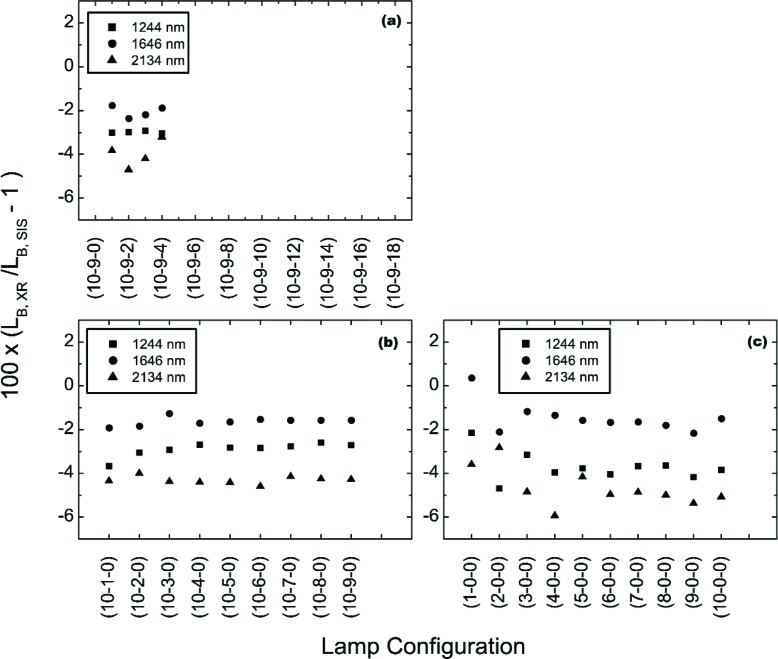
UA SWIR results of the off-axis measurements of the SIS100 for configurations where lamps of the same wattage were turned off sequentially. The measurements began on May 14 with 4 of the 200 W lamps, all of the 8 W lamps, and all of the 45 W illuminated, see (a); then individual 45 W lamps were extinguished, see (b); and then individual 8 W lamps were extinguished, see (c).

**Fig. 15:**
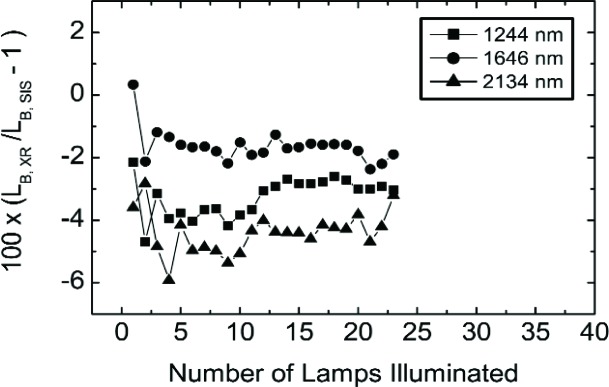
The results of Fig. 14 plotted as a function of number of lamps in the SIS100 that were illuminated for the measurements on May 14. The lines are to guide the eye. Configurations with one to nine 8 W lamps illuminated have abscissa values from 1 to 9; configurations with all 8 W and one to ten 45 W lamps illuminated have abscissa values from 10 to 19; and configurations with all 8 W, all 45 W and one to four 200 W lamps illuminated have abscissa values from 20 to 23.

Figure 14 shows the corresponding results for the shortwave infrared wavelengths in terms of three channels of the UA SWIR radiometer. The radiance levels are as in Fig. 12, except the UA SWIR instrument did not measure lamp configurations (10-9-18) to (10-9-5), so there are no measurements reported for the UA SWIR on May 15. In Fig. 15, the results are shown for three channels of the UA SWIR radiometer—1244 nm, 1646 nm, and 2134 nm. Contrary to the results in Fig. 13, the results in the shortwave near infrared exhibit no dependence on transitions in lamp configuration involving the extinction of the last lamp of a particular wattage. However, there is significant variability in the results for the dimmest levels compared to the brighter ones, see Fig. 14c.

### 5.3 SIS100 Repeatability and Stability

We use the term repeatability to describe the agreement of separate measurements of the SIS100, where “separate” denotes the SIS100 lamp configuration was changed, including turned off, or the transfer radiometer was moved or realigned, and so forth. We use the term stability to describe the temporal behavior of continuous measurements by a single radiometer. There are two estimators of stability: a) the results at a particular channel (wavelength) for a transfer radiometer during each on-axis measurement of the six principal levels (typical duration of between 5 min and 20 min); and b) the results at a particular channel (wavelength) for the EOS VXR and the UA SWIR during the off-axis monitoring of one of the six principal lamp configurations. This off-axis monitoring was possible only when these radiometers were not themselves measuring the SIS100. The EOS VXR recorded monitor data in its six channels while the UA SWIR recorded monitor data in its 1600 nm channel. Note that, for either repeatability or stability, the performance of the transfer radiometer as well as the SIS100 are equally important.

#### 5.3.1 Repeatabilty

**Fig. 16:**
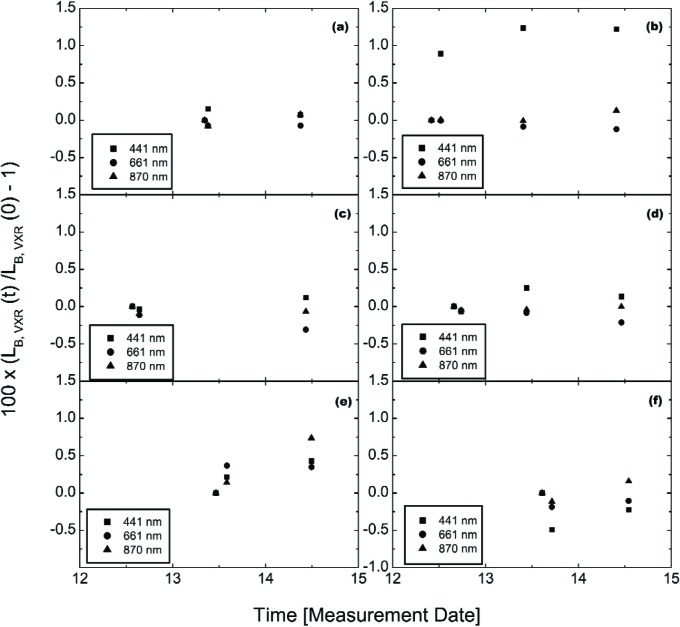
Repeatability of the SIS100 and EOS VXR measurement system for the six principal levels: (a) (10-9-11), (b) (10-9-4), (c) (10-9-0), (d) (10-5-0), (e) (10-0-11), and (f) (4-0-0). The EOS VXR results are normalized to the first measurement and plotted as a function of measurement time.

There are different types of measurements that speak to the repeatability. First, the EOS VXR measured each of the six principal levels twice, at the start and end of a measurement sequence for that particular lamp configuration. The measurements were made on-axis with the VXR moved in and out to accommodate the other transfer radiometers, without changing the SIS100. Second, the (10-9-4) and (10-5-0) configurations were measured twice on-axis by the EOS VXR and the UA SWIR. Third, during the off-axis studies on May 14, the six principal levels were measured by the EOS VXR and the UA SWIR. For three channels of the EOS VXR, these results are summarized in Fig. 16, where we have normalized the results to the initial measurement at that wavelength, and plotted the results as a function of time. The units on the abscissa are day in May, with time of day normalized to 24 h. Not shown in Fig. 16, but discussed in Sec. 5.2, the two off-axis measurements of the (10-9-14) configuration on May 14 and 15 indicated a discrepancy of up to 1.5 %.

The repeatability demonstrated in Fig. 16 using the VXR (labeled “2×” in Table 4) without turning off the SIS100 is within 0.25 %, except for the second measurement on May 12 at 441 nm, which was 0.9 % higher than the first, see Fig. 16b. Not shown, the corresponding result at 412 nm was even greater, about 1.3 %, but differences at 548 nm and 776 nm were small, about the same as those shown in Fig. 16b at 661 nm and 870 nm. The VXR monitor data at 441 nm, acquired in between the first and second on-axis measurements, shows a 0.33 % increase. The (10-9-4) configuration was the first measured in this comparison, and the VXR was the first instrument used. If we normalize the result at 441 nm to the second measurement on May 12, the first would be an outlier.

The (10-9-4) and (10-5-0) configurations were measured once by the EOS VXR on May 13, the day after the full study was performed (see Table 4). These results agree to within about 0.25 %, if the first measurement at 441 nm on May 12 of the (10-9-4) configuration is ignored, see above. Finally, the six principal levels were measured off-axis as described in Sec. 5.2. The agreement is good, again to within about 0.25 %.

#### 5.3.2 Stability

During the on-axis measurements of the six principal levels, the EOS VXR acquired data for about 6 min, except for the two dimmest levels, when this interval was doubled. This resulted in 8 or 15 samples at each measurement channel. The resulting relative standard deviations were between 0.03 % and 0.3 %; generally speaking the bluer measurement wavelengths and dimmer SIS100 radiances resulted in larger standard deviations.

**Fig. 17:**
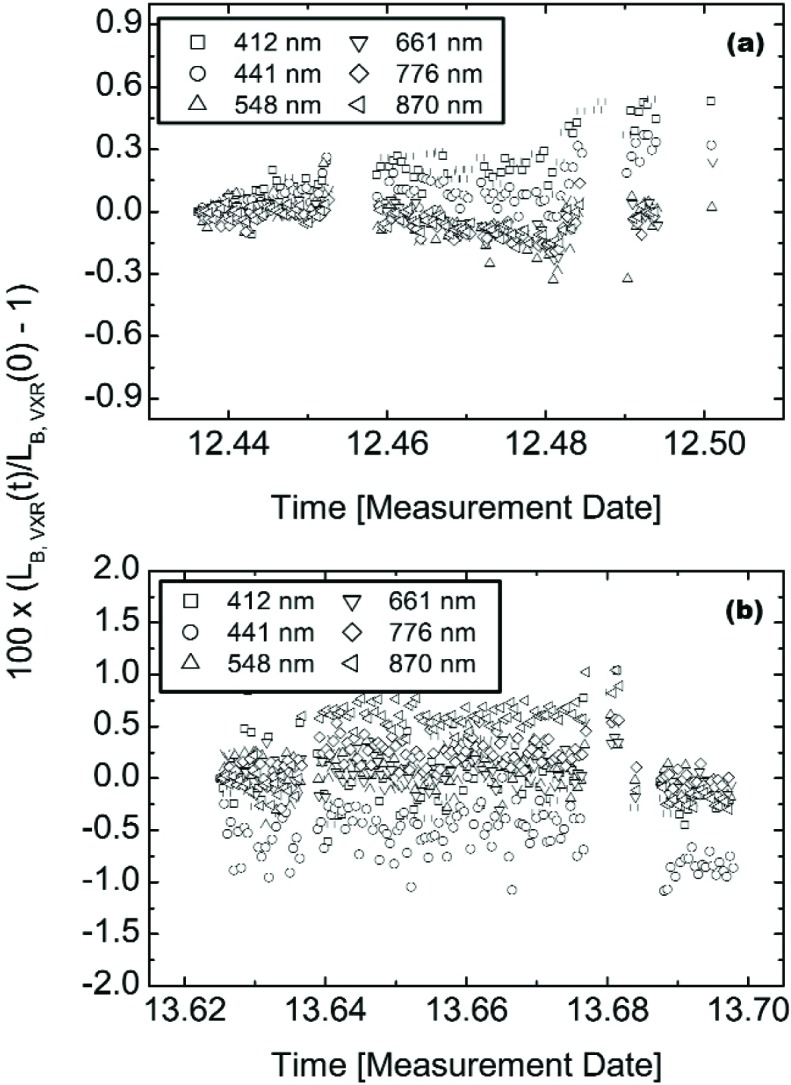
Typical results of the VXR as an off-axis monitor during the measurement of the six principal levels by the other radiometers for (a) lamp configuration (10-9-4) on May 12 and (b) lamp configuration (4-0-0) on May 13; note the scale change in the ordinate. All six channels of the VXR are shown as a function of the measurement date and time. For each channel, the results were normalized to the initial value and as plotted represent the change in percent.

During the off-axis monitoring of the six principal levels, the SIS100 was monitored for between 1.5 h and 2 h. Two examples are shown in Fig. 17 for the VXR monitoring of the (10-9-4) configuration on May 12 and the (4-0-0) configuration on May 13. The results are shown normalized to the first measurements as a function of time (same format as Fig. 16). The VXR was operated continuously, so spurious values that resulted during the movement of the other radiometers in and out of the on-axis measurement position have been removed. The relative standard deviations for these data sets, as well as all of the other results for the VXR monitoring of the six principal levels, are given in Table 7. The data for configuration (10-9-4) on May 13 shows that the SIS100 when properly warmed up is capable of exhibiting stabilities on the order of 0.08 % or better over several hours.

The data of Table 7 should be considered as upper limits to the true stability of the SIS100, because of possible failure to remove all spurious events, or the measurement uncertainty of the VXR itself. We compare the relative standard deviations for the on-axis VXR measurements, which arise from 6 min to 12 min measurement intervals, to those for the monitoring data sets given in Table 7, which arise from 1.5 h to 2 h measurement intervals. In some cases the relative standard deviations agreed well, for example at 412 nm and configuration (4-0-0) the values were 0.25 % and 0.29 % for the on- and off axis measurements, respectively. In other cases, the monitor data sets resulted in larger standard deviations, for example at 412 nm and configuration (10-9-4) on May 12 the values were 0.05 % versus 0.15 %. This was due to actual temporal drift, see the 412 nm and the 441 nm results in Fig. 17a or increased scatter, see Fig. 17b.

In principle, data from a monitor radiometer can be used to correct results to a common scale, if the monitor radiometer is very stable and used in a repeatable fashion. This type of correction has not been applied to the results of this comparison, but to demonstrate the feasibility, we fitted the monitoring data to a 5th order polynomial and report the maximum deviations in Table 7.

The EOS VXR monitor data indicated that the stability of the SIS100 for each sequentially measured sphere radiance level was a function of the preceding conditions. This was particularly true for blue wavelengths. For example, on May 12, the SIS100 was turned on at 07:11 PDT to lamp configuration (10-9-18). At the request of the comparison participants and 15 min before the beginning of the measurements of the various levels, the lamp configuration was changed (10-9-4). Over the next 105 min, the VXR detected a 0.53 % and 0.33 % increase in the radiance of the SIS100 at 412 nm and 441 nm, see Fig. 17a. For VXR wavelengths at 548 nm and above, the maximum radiance changes were on the order of only 0.06 %. On May 13, the (10-9-4) configuration was measured after the (10-9-11) configuration, 155 min after the sphere was initially turned on and 23 min following the level change. The VXR detected maximum radiance changes of less than 0.03 % for all six VXR channels.

During changes in the lamp configuration of the SIS100, the monitor data acquired by the VXR indicated that the radiance of the new sphere radiance level was realized to within 0.7 % of its final value within 1 min of the level change, this value being a complex function of the magnitude of the radiance change between levels and the wavelength. The final radiance value of the new radiance level was stabilized to within the measurement uncertainty of the VXR monitor data 8 min to 10 min after the level change. The SBRS practice of waiting 15 min after changing lamp configuration before calibrating or utilizing the SIS 100 is justified and supported by the VXR monitor data.

### 5.4 Calculation of Multiple Lamp Level Radiances

For the preflight calibration of Terra MODIS and the Landsat 7 ETM+, SBRS determined the radiances for multiple (wattage) lamp configurations from sums of measured radiances at individual (wattage) lamp configurations. For example, SBRS made measurements of the sphere output with ten 8 W lamps illuminated, level (10-0-0). SBRS also made measurements of the output with nine 45 W lamps illuminated (0-9-0) and with fourteen 200 W lamps illuminated (0-0-14). For SBRS, the SIS100 radiance at lamp configuration (10-9-14) is calculated as the sum of the radiances from the three measurements.

On May 15, measurements of the SIS100 were made to verify this superposition method used by SBRS. Measurements were made by the EOS VXR and the UA SWIR at several lamp configurations involving lamps of identical wattage. These results were used to calculate the radiances at several multiple lamp levels. These calculated radiances were compared with actual measurements at the multiple lamp levels.

**Fig. 18:**
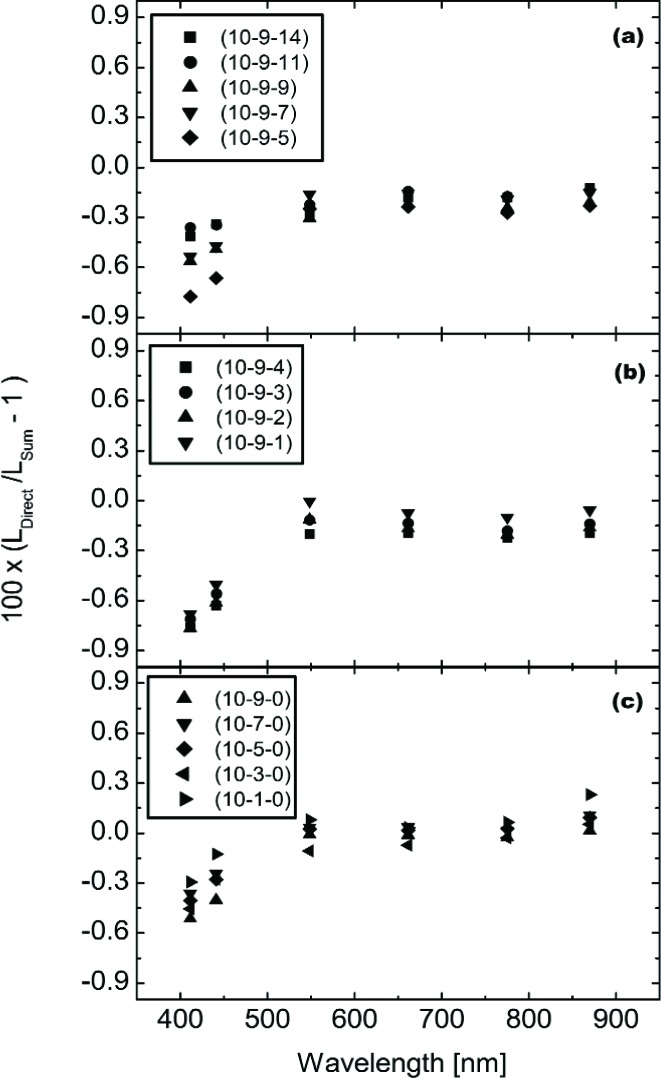
Comparisons, using the EOS VXR, of the direct measurements of lamp configurations with more than one lamp wattage type and the sum of separate measurements of the corresponding configurations with only lamps of a similar wattage illuminated. The results are shown as percent difference versus VXR wavelength for (a) configurations (10-9-14) to (10-9-5), (b) configurations (10-9-4) to (10-9-1), and (c) configurations (10-9-0) to (10-1-0).

For the EOS VXR, Fig. 18 shows the comparison of the calculated radiances (using the summed single wattage lamp configurations measured by the VXR) with the multiple wattage lamp configurations measured by the VXR. The results are shown as the differences of the summed values from the measured values at each EOS VXR wavelength. In all cases, the disagreement between the calculated and measured values is less than 1 %. Except for the wavelengths in the blue (412 nm and 441 nm), the disagreement is less than 0.5 %.

**Fig. 19:**
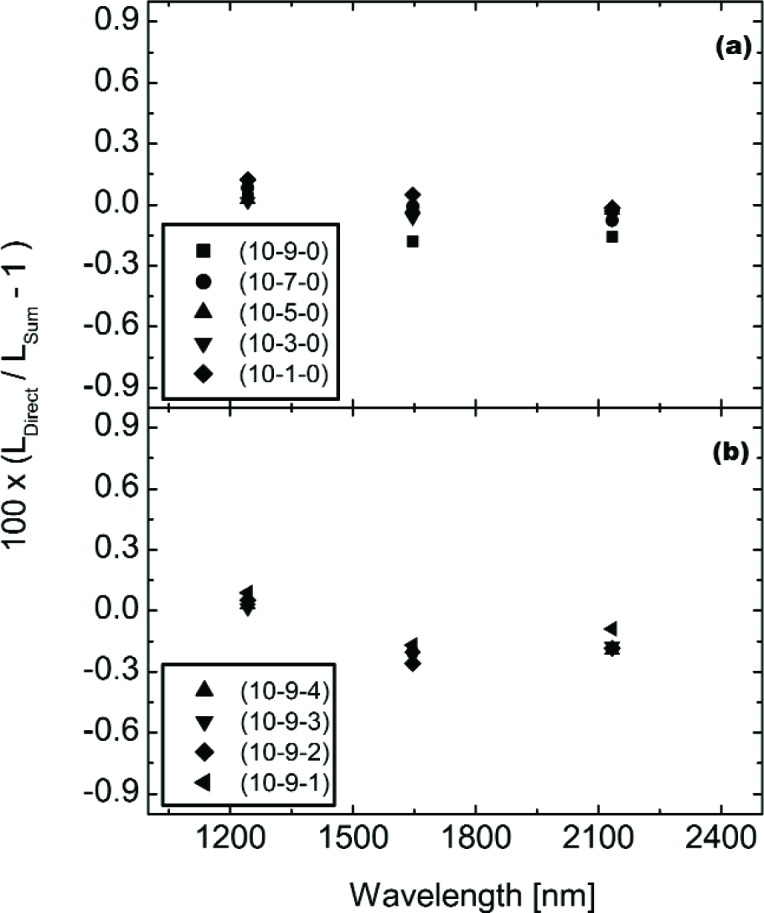
Comparisons, using the UA SWIR, of the direct measurements of lamp configurations with more than one lamp wattage type and the sum of separate measurements of the corresponding configurations with only lamps of a similar wattage illuminated. The results are shown as percent difference versus UA SWIR wavelength for (a) configurations (10-9-4) to (10-9-1), and (b) configurations (10-9-0) to (10-1-0).

Figure 19 shows the results for three channels of the UA SWIR (1244 nm, 1646 nm, and 2134 nm). The differences in Fig. 14 are calculated in the same manner as those in Fig. 18 for the EOS VXR. In the shortwave infrared, the difference between the calculated and measured values is less than 0.5 % at all levels.

## 6. Concluding Remarks

The primary goals of the May 1998 radiometric measurement comparison at Raytheon SBRS were to independently measure the radiance of the SIS100 and to validate the uncertainties assigned by SBRS to these values. In the visible and near infrared, the results of comparing the SBRS assigned radiance scale with the measurements of the VXR, UA VNIR, and LXR radiometers indicated that the SBRS radiances agreed with the values determined by the transfer radiometer measurements to within ±3.0 %. In the shortwave infrared, the corresponding result using the EOS SWIXR and the UA SWIR was to within ±4.0 % for wavelengths not affected by atmospheric absorption, e.g., from 850 nm to 1300 nm, 1540 nm to 1800 nm, and 2050 nm to 2270 nm. The latter region, at the longest wavelengths, resulted in the largest discrepancies; this spectral region corresponds to MODIS band 7 at 2130 nm. Overall, the SBRS and transfer radiometer measurements agreed to within their combined expanded (*k* = 2) measurement uncertainties. In conclusion, we have validated the SBRS goal of calibrating the SIS100 to ±3.0 % in the visible and near infrared spectral region.

In regions of strong atmospheric absorption, e.g, from 1300 nm to 1540 nm and 1700 nm to 2100 nm, the discrepancies between SBRS and the transfer radiometers are large, up to 24 % for the EOS SWIXR. Atmospheric absorption affects both the SIS100 and the transfer radiometers, and we draw no conclusions here from the magnitude of the observed differences. However, the impact for MODIS is for the uncertainty assessment for band 26 at 1375 nm, here, the uncertainties estimated by SBRS appear to be underestimated, see Table 2. Numerical interpolation is also an issue in these spectral regions, because NIST does not typically provide values of spectral irradiance or BRF to radiometric source standards in regions of atmospheric absorption.

Part of the uncertainty assessment involved examining the repeatability and stability of the SIS100. The repeatability of the SIS100 was determined to be within 0.25 % from measurements made by the VXR, but there were outliers such as the May 14 and 15 measurements of the (10-9-14) lamp configuration. The stability of the SIS100 was monitored by the VXR and UA SWIR. The SIS100, when afforded sufficient warm up time, e.g. 15 min, exhibited stability of 0.08 % or better.

The linearity characterizations performed for MODIS and ETM+ by SBRS utilized the SIS100 over its different radiance levels. Because the transfer radiometers used in this comparison are linear to 0.25 % or better, the measurements over the range of radiance levels using these instruments indicates the radiances assigned by SBRS to the SIS100 for this comparison activity are in the correct proportion to within about 1 %, except for the dimmest level, configuration (4-0-0), and then only at 2250 nm.

A secondary goal of the May 1998 radiometric comparison was to compare SIS100 radiances determined by addition of the radiances observed with either 200 W, 45 W, or 8 W lamps illuminated and comparison to the radiance observed with the corresponding multiple wattage configuration. For all levels examined, the disagreement was less than 1 %. This result effectively and retrospectively validated Raytheon SBRS’s calibration technique for the SIS100 for the MODIS instruments on the EOS Terra platform.

The comparison afforded an opportunity to compare the transfer radiometers. The first result was that different radiometers from the same institute gave consistent results in the spectral regions where they could be compared. The second result was measurements of the separate SIS100 radiance levels, to the extent that the SIS100 was stable, allow us to compare all of the radiometers, and hence the accuracy of their radiometric calibration, for the overlap spectral regions. All of these results agree to within the combined expanded uncertainty (*k* = 2).

## Figures and Tables

**Table 1:** Specifications of the SBRS Spherical Integrating Source (SIS100)

Inner diameter	100 cm
Aperture diameter	45.72 cm
Aperture thickness	0.3175 cm
Coating on inner wall	Barium sulfate
Lamp configuration (possible number, rated wattage and voltage)	eighteen 200 W and 30 V nine 45 W and 7 V ten 8 W and 5 V
Sphere operation	Lamp current is regulated to 0.03 % Lamps are wired in series
Thermal cooling	Air circulation with fan
Internal monitor detector	None

**Table 2:** Relative combined standard uncertainty for the spectral radiance calibration of the SIS100 as calculated by SBRS for three spectral regions. The notation B7 and B26 refers to MODIS bands 7 (2.13 µm) and 26 (1.375 µm)

Description	VIS	NIR	SWIR
Irradiance standard	0.5 %	0.5 %	0.6 %
Diffuse reflectance target	0.8 %	0.8 %	1.0 %
Standard lamp usage (subtotal)	0.9 %	0.8 %	0.8 %
orientation	0.3 %	0.3 %	0.3 %
distance	0.5 %	0.5 %	0.5 %
current	0.4 %	0.3 %	0.2 %
scattered light	0.5 %	0.5 %	0.5 %
Spectroradiometer (subtotal)	1.7 %	1.3 %	1.3 %
wavelength calibration	0.3 %	< 0.1 %	< 0.1 %
polarization	< 0.1 %	< 0.2 %	0.5 %
finite slit width	0.1 %	< 0.1 %	< 0.1 %
linearity	1.5 %	1.0 %	1.0 %
meas. unc. (standard lamp)	0.5 %	0.5 %	0.5 %
meas. unc. (SIS(100))	0.5 %	0.5 %	0.5 %
SIS(100) (subtotal)	0.8 %	0.8 %	1.6 %
current	0.4 %	0.3 %	0.2 %
nonuniformity	0.7 %	0.7 %	0.7 %
atmospheric absorption	< 0.1 %	< 0.1 %	1.3 % (B26)
interpolation	< 0.2 %	< 0.2 %	0.6 % (B7 & B26)
SIS(100), combined	2.3 %	2.0 %	2.5 %

**Table 3:** Wavelengths and bandwidths of the radiometers used to measure the SIS100 in May 1998. Except for the EOS SWIXR, all are filter radiometers with fixed spectral shapes, and the values are in order of the channel number. The EOS SWIXR is a double monochromator. In May 1998, it made sets of measurements with 29 nearly equal steps from 850.2 nm to 2247.9 nm and with bandwidths that decreased gradually with increasing wavelength from 16.5 nm to 12.5 nm

EOS VXR		UA VNIR		GSFC LXR		UA SWIR		EOS SWIXR	
*λ* (nm)	Δ*λ* (nm)	*λ* (nm)	Δ*λ* (nm)	*λ* (nm)	Δ*λ* (nm)	*λ* (nm)	Δ*λ* (nm)	*λ* (nm)	Δ*λ* (nm)
411.8	10.8	412.8	14.9	480.7	61.1	746.9	10.5	850.2	16.5
441.0	10.5	441.8	11.8	560.7	76.5	868.7	12.1	900.4	16.5
548.4	10.2	488.0	9.6	662.3	60.3	940.0	16.2	↑ every 50 nm ↓	
661.4	9.5	550.3	9.9	827.0	109.4	1243.5	16.1		
775.5	11.1	666.6	9.9	440.0	10.4	1380.8	27.7		
870.0	13.4	746.9	10.6	661.1	9.4	1646.0	23.0		
		868.1	14.0			2133.5	25.2	2197.9	12.5
						2164.2	20.7	2247.9	12.5
						2207.8	22.1		
						2263.0	23.5		

**Table 4:** Measurement schedule at SBRS for the six principal radiance levels of the SIS100, with the radiometers on-axis. If the VXR was used twice, denoted “2×,” these measurements were at the start and the end of that lamp configuration. Otherwise, the radiometers were used once, denoted “1×”

	May 12, 1998					May 13, 1998				
SIS100 Level	EOS VXR	GSFC LXR	UA VNIR	EOS SWIXR	UA SWIR	EOS VXR	GSFC LXR	UA VNIR	EOS SWIXR	UA SWIR
(10-9-11)						2×	1×	1×		
(10-9-4)	2×	1×	1×	1×	1×	1×				1×
(10-9-0)	2×	1×	1×	1×	1×					
(10-5-0)	2×	1×	1×	1×	1×	1×				1×
(10-0-0)						2×	1×	1×	1×	1×
(4-0-0)						2×	1×	1×	1×	1×

**Table 5:** Comparisons with the SBRS radiances for the SIS100 at common wavelengths for the UA visible and shortwave infrared radiometers. The table gives the percent differences of the radiometer measurements from the band averaged spectral radiances calculated from the SBRS spectral radiance values as explained in the text

SIS100 Level	Difference from SBRS (%)			
	UA VNIR		UA SWIR	
	746.9 nm	868.1 nm	746.9 nm	868.7 nm
(10-9-4)	1.69	0.95	0.88	−1.32
(10-9-0)	0.51	0.79	−0.12	−1.28
(10-5-0)	0.75	−0.16	−0.09	−2.28
(10-0-0)	−0.21	−0.85	−1.15	−2.59
(4-0-0)	−0.90	−1.28	−1.45	−2.98

**Table 6:** Comparisons with the SBRS radiances for the SIS100 at neighboring wavelengths for the EOS visible and shortwave infrared radiometers. The table gives the percent differences of the radiometer measurements from the band averaged spectral radiances calculated from the SBRS spectral radiance values as explained in the text. The EOS SWIXR made no measurements at the VXR wavelength of 870 nm, so the measurements from the two closest SWIXR wavelengths are shown. The SWIXR was designed for measurements at 900 nm and longer

SIS100 Level	Difference from SBRS (%)		
	EOS VXR	EOS SWIXR	
	869.9 nm	850.2 nm	900.4 nm
(10-9-4)	1.32	1.33	1.30
(10-9-0)	1.07	0.88	1.65
(10-5-0)	0.01	−0.30	0.54
(10-0-0)	−0.71	−2.16	−1.08
(4-0-0)	−1.17	−7.45	−2.54

**Table 7:** Stability of the SIS100. The top table values represent the maximum deviations in percent of a 5th order polynomial fit to the EOS VXR monitor signals. The bottom table values represent the standard deviations from the mean in percent of the monitor radiometer signals

Wavelength (nm)	May 12 Lamp Configurations			(10-9-11)	May 13 Lamp Configurations			(4-0-0)
	(10-9-4)	(10-9-0)	(10-5-0)		(10-9-4)	(10-5-0)	(10-0-0)	
411.7	±0.24 %	±0.08 %	±0.14 %	±0.13 %	±0.03 %	±0.18 %	±0.08 %	±0.15 %
	0.15 %	0.08 %	0.10 %	0.09 %	0.04 %	0.19 %	0.23 %	0.29 %
441.0	±0.17 %	±0.09 %	±0.12 %	±0.14 %	±0.02 %	±0.20 %	±0.20 %	±0.25 %
	0.10 %	0.08 %	0.09 %	0.08 %	0.05 %	0.16 %	0.26 %	0.26 %
548.4	±0.07 %	±0.08 %	±0.10 %	±0.08 %	±0.04 %	±0.27 %	±0.20 %	±0.17 %
	0.10 %	0.07 %	0.08 %	0.07 %	0.07 %	0.14 %	0.19 %	0.18 %
661.4	±0.07 %	±0.06 %	±0.08 %	±0.07 %	±0.04 %	±0.13 %	±0.11 %	±0.14 %
	0.07 %	0.06 %	0.07 %	0.05 %	0.04 %	0.10 %	0.15 %	0.16 %
775.5	±0.06 %	±0.05 %	±0.14 %	±0.05 %	±0.04 %	±0.14 %	±0.11 %	±0.19 %
	0.06 %	0.06 %	0.11 %	0.04 %	0.04 %	0.10 %	0.14 %	0.17 %
869.9	±0.06 %	±0.2 %	±0.33 %	±0.06 %	±0.03 %	±0.11 %	±0.45 %	±0.52 %
	0.05 %	0.18 %	0.32 %	0.04 %	0.04 %	0.09 %	0.39 %	0.37 %

## Footnotes

1 Certain commercial equipment, instruments, or materials are identified in this paper to foster understanding. Such identification does not imply recommendation or endorsement by the National Institute of Standards and Technology or the National Aeronautics and Space Administration, nor does it imply that the materials or equipment are necessarily the best available for the purpose.

2 A trap detector is an arrangement of three to six photodiodes (typically silicon) arranged geometrically so reflections from one photodiode are directed to the subsequent photodiode in the system.

3 The linearity of radiometer output versus spectral radiance of the SIS100 for visible and near infrared filter radiometers such as the EOS VXR, the UA VNIR, and the GSFC LXR that are constructed using silicon photodiodes and well understood transimpedance amplifiers is expected to be about 0.1 %, based on instrument design, characterization, and previous experience [12].

4 The nonlinearity of the UA SWIR was measured to be less than 0.25 % for signal levels corresponding to between 20 % and 80 % of the full-scale range of the lock-in amplifier [20].
